# Enhanced photocatalytic activity of Se-doped TiO_2_ under visible light irradiation

**DOI:** 10.1038/s41598-018-27135-4

**Published:** 2018-06-08

**Authors:** Wei Xie, Rui Li, Qingyu Xu

**Affiliations:** Yangzhou Polytechnic Institute, Yangzhou, Jiangsu China

## Abstract

Anatase TiO_2_ is a typical photocatalyst, and its excellent performance is limited in ultraviolet light range due to its wide band gap of 3.2 eV. A series of Se-doped TiO_2_ nanoparticles in anatase structure with various Se concentrations up to 17.1 at.% were prepared using sol-gel method. The doped Se ions are confirmed to be mainly in the valence state of + 4, which provides extra electronic states in the band gap of TiO_2_. The band gap is effectively narrowed with the smallest gap energy of 2.17 eV, and the photocatalytic activity is effectively improved due to the extended absorption range. The photocatalytic activity was evaluated by the degradation of Rhodamine B (RhB) in aqueous solution under visible light irradiation. The results show that Se doping significantly improves the photocatalytic activity of TiO_2_ and 13.63 at.% Se-doped TiO_2_ has the best performance.

## Introduction

In recent years, global environmental problems are becoming more and more concerned due to the severe pollutions, especially organic pollutants. A new solution for the degradation of pollutants is photocatalysis which is very effective and environmental friendly, compared with many other methods. Common photocatalysts usually include metal oxides and sulfides, such as, TiO_2_, ZnO, SnO_2_, ZrO_2_, CdS, and so on. Among them, the first discovered photocatalyst, TiO_2_, has been intensively studied^1,2^. However, the band gap of TiO_2_ is 3.2 eV for anatase phase and 3.0 eV for rutile phase, respectively. Thus, TiO_2_ is nearly inactive under visible light irradiation so that sustainable solar energy can’t be used adequately.

Many efforts have been devoted to overcome the shortcomings and enhance the photocatalytic properties of TiO_2_, especially in the visible light range. For example, ion doping^3–7^, anatase-rutile phase coexisting^8^, or p-n heterojunction formation^9^, were explored. Among them, ion doping was an efficient approach^10^. Many elements were doped into TiO_2,_ such as Ag^11^, Fe^12,13^, N^14–16^, S^17^, C^18–20^, and B^21^, etc. New energy levels can be introduced by the doping ions to decrease the band gap energy and enhance the photocatalytic activity of TiO_2_, especially under visible light irradiation. Furthermore, electron-hole recombination rate can be effectively reduced due to the formation of charge trapping sites by foreign ions^22,23^.

Selenium (Se) is in the same group element with oxygen (O) in the periodic table of elements. Se possesses cationic (Se^4+^, Se^6+^) and anionic (Se^2−^) characters, which was suggested to introduce extra electronic states in the band gap by calculations^24^. However, TiO_2_ doped with Se has not been widely studied. Zhang *et al*. prepared the SeO_2_/TiO_2_ nanocomposites, in which the recombination of electron-hole pairs might be effectively suppressed due to the formation of heterostructure and absorption might be extended to visible light due to the narrow band gap of 2.0 eV of Se° by the reduction of Se^4+^ ^25^. Se was doped into anatase TiO_2_ by various methods, with narrowed band gap energy and effectively extended absorption to the visible light range. Therefore, significantly enhanced photocatalytic activity was achieved under visible irradiation^24,26–28^. However, the Se doping concentration was rather low, due to the easy sublimation (315–317 °C) of SeO_2_ in the calcining processes^28^. To further narrow the band gap and improve the photocatalytic activity under visible light irradiation, higher concentration of Se should be doped into TiO_2_. In this paper, we significantly increase the doping concentration of Se up to 17.1 at.% in TiO_2_ by lowering the calcining temperature. The band gap is effectively narrowed and the photocatalytic activity under visible light irradiation is improved.

## Experimental

### Sample preparation

The chemicals used in this study were absolute ethanol (C_2_H_5_OH), tetrabutyl titanate (C_16_H_36_O_4_Ti), nitric acid (HNO_3_), selenium dioxide (SeO_2_), which were of analytical grade and used as received without further purification. The distilled water was produced using a Direct-Q Millipore filtration system with resistivity of 18.2 MΩ·cm (Millipore Limited, Watford, UK).

A series of Se-doped TiO_2_ nanoparticles with various Se concentration were prepared by sol-gel method. The Se-doped TiO_2_ powders were synthesized as following steps: solution A was the mixture of C_16_H_36_O_4_Ti and C_2_H_5_OH which was stirred to transparent solution in dropping process of nitric acid, solution B was ethanol solution of different concentration of SeO_2_. The amount of SeO_2_ used was based on the designed atomic concentration of Se in TiO_2_ (Ti_1−x_Se_x_O_2_), which were 0 at.% (pure TiO_2_), 5 at.%, 10 at.%, 15 at.%, 20 at.% and 25 at.%, respectively. Solution B was dropped into solution A by violent stirring. The uniform sol solution was kept at room temperature for 16 h till it became a gel solution. The gel solution was dried at 120 °C until the xerogel was generated, then the xerogel was calcined at 300 °C for 3 h to get the final powders. The obtained products are denoted as TiO_2_, TSe5, TSe10, TSe15, TSe20, TSe25, respectively, and the number denotes the designed atomic percentage of Se in Ti_1−x_Se_x_O_2_.

### Structural characterization

The crystal structure of Se-doped TiO_2_ nanoparticles was analyzed by X-ray diffraction (XRD, Rigaku SmartLab3) using Cu Kα radiation (λ = 0.15418 nm). The UV-Vis diffuse reflectance spectra (DRS) were recorded using a UV-visible spectrophotometer (JASCO, UV-670) with a wavelength range of 200–800 nm. The photoluminescence (PL) and PL excitation (PLE) spectra were recorded on a PL spectrofluorometer (HJY-FL3–211-TCSPC) at an excitation wavelength of 365 nm. The morphologies were studied by a scanning electron microscope (SEM, FEI Inspect F50), equipped with an energy dispersive X-ray spectroscope (EDX). EDX was performed for the chemical analysis of the doped samples, and the measured concentrations are listed in Table 1. As can be seen, the measured concentrations of Se are smaller than the designed values, which might be due to the partial sublimation of SeO_2_ during the calcining processes^28^. Raman studies were carried out on Raman spectrometer (Horiba Jobin Yvon Lab RAM HR 800) under the backscattering geometric configuration at room temperature. The valence states of each element were studied by X ray photoelectron spectroscopy (XPS, Thermo Fisher Scientific) with Al Kα X ray source (hν = 1486.6 eV). Binding energies were referenced to the C1s peak at 284.5 eV.

**Table 1:** Lattice constant *a* and *c*, Crystallite size, Band gap and EDX determined Se concentration of Se-doped TiO_2_.

Catalyst powder	*a* (nm)	*c* (nm)	Crystallite size (nm)	Band gap (eV)	EDX Se (at.%)
TiO_2_	3.801	9.542	7.4	2.82	0
TSe5	3.793	9.505	10.9	2.39	2.2
TSe10	3.800	9.617	7.7	2.19	6.76
TSe15	3.789	9.567	6.8	2.73	11.77
TSe20	3.789	9.567	6.4	2.58	13.63
TSe25	3.789	9.592	6.3	3.03	17.1
Degussa P25				3.04	0

### Photocatalytic experiments

The visible-light-induced photocatalytic activity was evaluated in aqueous solution, by using Rhodamine B (RhB, Tianjin China Chemical Reagent Ltd.) as a model contaminant in this study. The light irradiation system contained a 500 W Xe lamp (Beijing Trusttech Co Ltd, CHF-XM), equipped with visible light pass filter (400–800 nm). In photocatalytic experiment: 0.4 g photocatalyst was mixed with a 150 mL RhB solution (7.5 mg·L^−1^), an adsorptive experiment primarily was proceeded in the dark for 30 min to achieve absorption-desorption equilibrium of RhB onto the catalyst surface before light irradiation. After interval of scheduled time, 2–3 mL RhB solution was collected for concentration analysis, which was monitored by using a UV-vis spectrophotometer (Hitachi U-3900).

## Results and Discussion

**Figure 1:**
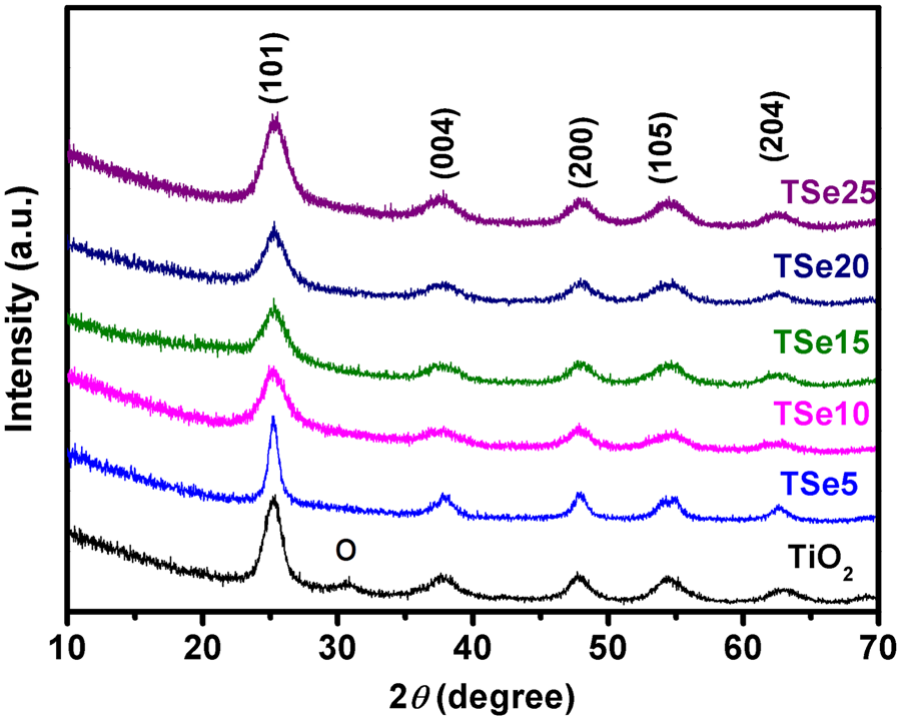
XRD patterns of Se-doped TiO_2_. “o” labels the diffraction peak from orthorhombic phase.

Figure 1 shows the XRD patterns of TiO_2_ and Se-doped TiO_2_ powders. As can be seen, the main diffraction peaks of TiO_2_ powders can be indexed to anatase phase (PDF No. 21–1272), except for a small peak labeled by “o”, indicating a small concentration of orthorhombic TiO_2_ phase (PDF No. 65–2448). Interestingly, by doping with Se, the diffraction peak of orthorhombic phase disappears, and only anatase phase can be observed, suggesting that Se doping might promote the formation of anatase phase. During the polycondensation process, less TiO_6_ octahedra units were formed due to the doping of Se. Thus, there was increased dispersion among the TiO_6_ octahedra units and as a result the building units shared their corners and anatase phase formation took place^27^. Furthermore, no Se-related oxides or other impurities can be observed. This suggests the uniform distribution of Se ions without aggregation in doped-TiO_2_. The lattice constants *a* and *c* are calculated from the XRD patterns, and summarized in Table 1. As can be seen, with 5% Se doping, the lattice constants *a* and *c* decrease, and both are smaller than those of TiO_2_, which is due to the smaller radius of Se^4+^ (0.64 Å) than Ti^4+^ (0.745 Å) (The valence state of Se is determined to be + 4 by XPS, which will be discussed later)^29^. With further increasing Se doping concentration, the lattice constant *a* is still smaller than that of TiO_2_, but lattice constant c becomes larger than that of TiO_2_. The effect of Se doping on the crystallite size can be evaluated according to the Scherrer’s equation, *D* = K*λ*/*β*cos*θ*, where K is the constant depending how the half height width of selected diffraction peak is determined (here we use 0.89), *λ* is the wavelength of X ray (0.15418 nm), *β* is the half height width, and *θ* is the Bragg angle. Here we select (101) peak to estimate the crystallite size, which is the strongest diffraction peak for all the samples. As can be seen, the peak becomes narrower first with small doping concentration of TSe5, and then becomes broader with further increasing the doping concentration. The calculated crystallite size is listed in Table 1. Similar phenomenon was observed by Khan *et al*., though the doping concentration was much smaller in their work^27^. Due to the small concentration of Se in TSe5, the less formation of TiO_6_ octahedra might facilitate the polycondensation process of TiO_6_ octahedra in corner sharing arrangement, leading to the larger crystallite size of anatase phase^27^. However, with further increasing the doping concentration, the doped Se ions might not occupy the lattice site, and thus the growth of the crystallite size was prevented^30^. The similar rule but much higher Se concentration in our work compared with Khan *et al*.’s work might be due to the much lower calcining temperature in our work, leading to the much smaller crystallite size and much higher tolerable doping concentration^27^. The morphologies of the samples are further studied by SEM, and the images are shown in Fig. S1 (Supplementary materials). The particle sizes of all samples are all smaller than 10 nm, which is consistent with XRD results.

**Figure 2:**
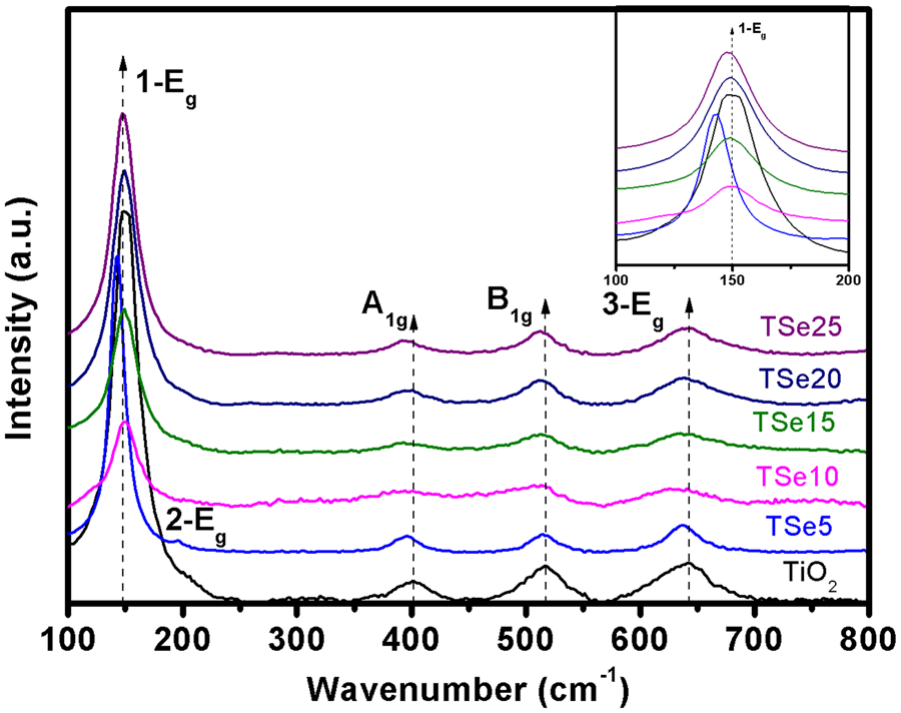
Raman spectra of Se-doped TiO_2_, inset shows the enlarged view of 1-E_g_ peak.

Raman spectroscopy is a powerful technique for the investigation of crystalline and defect structure, and to elucidate the various phases of TiO_2_^27^. The Raman spectra of Se-doped TiO_2_ with various Se concentrations are shown in Fig. 2. The peak positions are observed at 147 cm^−1^ (1-E_g_), 398 cm^−1^ (A_1g_), 514 cm^−1^ (B_1g_), 639 cm^−1^ (3-E_g_) for TiO_2_, corresponding to the vibration modes of anatase phase^27^. All the Se-doped TiO_2_ exhibit approximately the same bands, which is consistent with the pure anatase phase observed by XRD. TSe5 shows an extra peak at 195 cm^−1^, which corresponds to the 2-E_g_ band. This confirms the better crystalline structure of TSe5. The Se doping effect can be further evaluated from the shape and position of Raman peak, as shown the enlarged view of 1-E_g_ band in inset of Fig. 2. The peak shows the general tendency of becoming broader and shifting of the peak position to smaller wavenumbers, indicating the increasing distortion and smaller crystallite size with increasing Se doping concentration^31^. Interestingly, TSe5 shows a distinct behavior, with much narrower peak and significant shift to smaller wavenumbers. This can be understood by the better crystalline structure with much larger crystallite size for TSe5.

**Figure 3:**
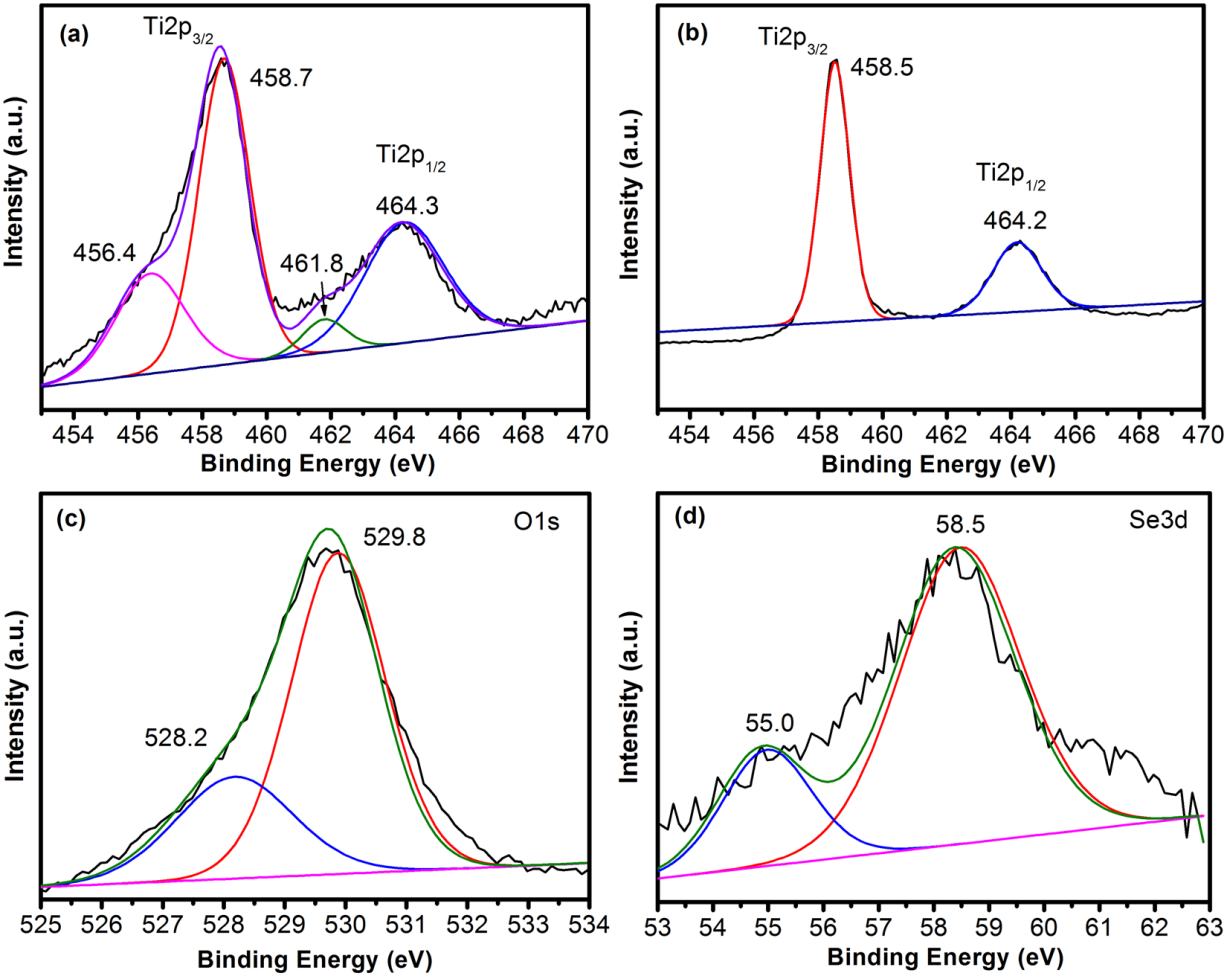
XPS spectra of (**a**) Ti2p, (**c**) O1s, and (**d**) Se3d for TSe20. (**b**) XPS spectrum of Ti2p for TiO_2_ annealed at 500 °C for 1 hour.

XPS was used to study the chemical states and electronic structure of Se-doped TiO_2_. Figure 3 shows the XPS spectra of Ti, O and Se for TSe20. The Ti2p3/2 and Ti2p1/2 peaks can be deconvoluted into two sets of peaks, the stronger peaks at 458.7 eV and 464.3 eV correspond to Ti^4+^, confirming the main valence state of + 4 for Ti in Se-doped TiO_2_^6^. A set of weaker peaks at 456.4 eV and 461.8 eV can be attributed to the Ti^3+^^6^. This might be due to the low calcining temperature, oxygen is not active enough to fully oxidize Ti, resulting in the formation of O vacancies and Ti^3+^ sites in TiO_2_ lattice^32^. To confirm the formation of Ti^3+^, we further annealed the TiO_2_ powders at 500 °C for 1 hour, and the XPS spectrum is shown in Fig. 3(b). As can be seen, only Ti2p3/2 peak at 458.5 eV and Ti2p1/2 peak at 464.2 eV can be observed, indicating the valence state of + 4. In Fig. 3(c), O1s peak can be deconvoluted into two peaks, one at 529.8 eV, and the other at 528.2 eV_._ The peak at 529.8 eV is attributed to the Ti-O bond^32^. The observation of peak at 528.2 eV is quite abnormal, since the bonding energy of Se-O in SeO_2_ should be higher. It is known that the O2p peak in La_2_O_3_ is 528.8 eV, which is due to the higher ionic nature of the La-O bonding^33^. Thus O ions will have lower binding energy when they attract more electrons. In the Se-doped TiO_2_ with much higher Se concentration, Se may not occupy the lattice sites due to the large lattice distortion. O may attract electrons not only from the neighboring Ti, but also from Se ions due to the formation of Ti-O-Se structure, leading to lower bonding energy, in comparison with O ions locating at regular lattice site^34^. Figure 3(d) shows the Se3d XPS peak, which can be deconvoluted into two peaks: one at 58.5 eV, and the other at 55.0 eV. The peak at 58.5 eV can be attributed to Se^4+^, and the slightly smaller value might be due to the doping in TiO_2_ lattice environment^27^. The peak at 55 eV indicates the existence of small amount of Se^0^ ^26^, which might be due to the reduction from C and H in the sources and further confirms the less chemical activity of O at such low calcining temperature.

**Figure 4:**
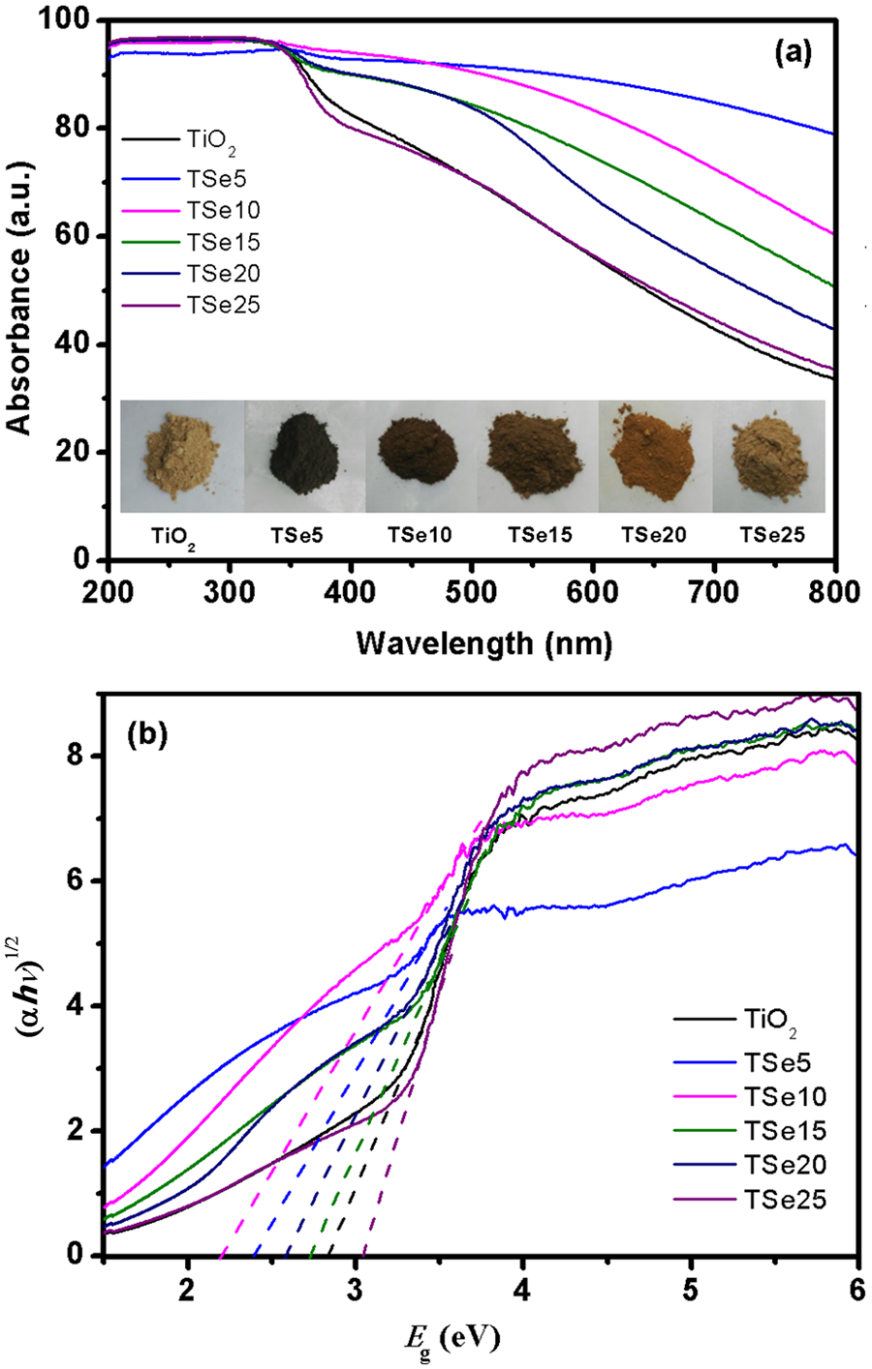
(**a**) UV-vis DRS of Se-doped TiO_2_. Insets show the corresponding photos of Se-doped TiO_2_ powders, (**b**) Curve-fitting by using the Kubelka-Munk function method for the absorption curves.

To investigate the optical absorption properties of Se-doped TiO_2_ nanoparticles, the UV-vis DRS were recorded and the results are shown in Fig. 4. The absorption edges of TiO_2_ shift to longer wavelength in the visible region, which is consistent with its brown-colored appearance, as shown in the inset of Fig. 4(a). This can be understood by the existence of Ti^+3^ due to the large concentration of O vacancies and disorder in TiO_2_ with very low calcining temperature^35,36^. With Se doping, the TiO_2_ powders first exhibits darker color, with the darkest color for TSe5. Then the color becomes pale with further increasing the Se doping concentration. TSe5 shows the highest absorption coefficient in the visible range, which continues to decrease with further increasing Se doping concentration. TSe25 shows almost the same absorption coefficient as TiO_2_. The optical band gap of Se-doped TiO_2_ can be evaluated by its absorption spectrum. The optical absorption near the band edge of a semiconductor often obeys the Kubelka-Munk equation: (α*hν*)^n^ = A(*hν* − *E*_g_), where A is a constant, *hv* is corresponding to photon energy, *E*_g_ is the band gap of the semiconductor, α is the absorption coefficient, and n is 0.5 for indirect band gap materials, such as TiO_2_^37,38^. The approximate values of the band gap can be obtained from the intercept of the tangent to X-axis, as shown in Fig. 4(b). The calculated band gap values are listed in Table 1. As can be seen, TSe10 shows the smallest *E*_g_ of 2.19 eV. There are two competing effects which may influence the final *E*_g_ of Se-doped TiO_2_. The Se doping may introduce additional electronic energy levels inside the band gap, which will effectively narrow the band gap^24^. The suppressed PLE peaks from TiO_2_ host lattice, shown in Fig. S2 (Supplementary materials), give an indirect evidence for the introduction of extra energy levels from doped Se ions. However, with further increasing Se concentration higher than that of TSe5, the crystallite size becomes smaller. The band gap increases with a decrease in crystallite size due to the quantum size effect^27^.

**Figure 5:**
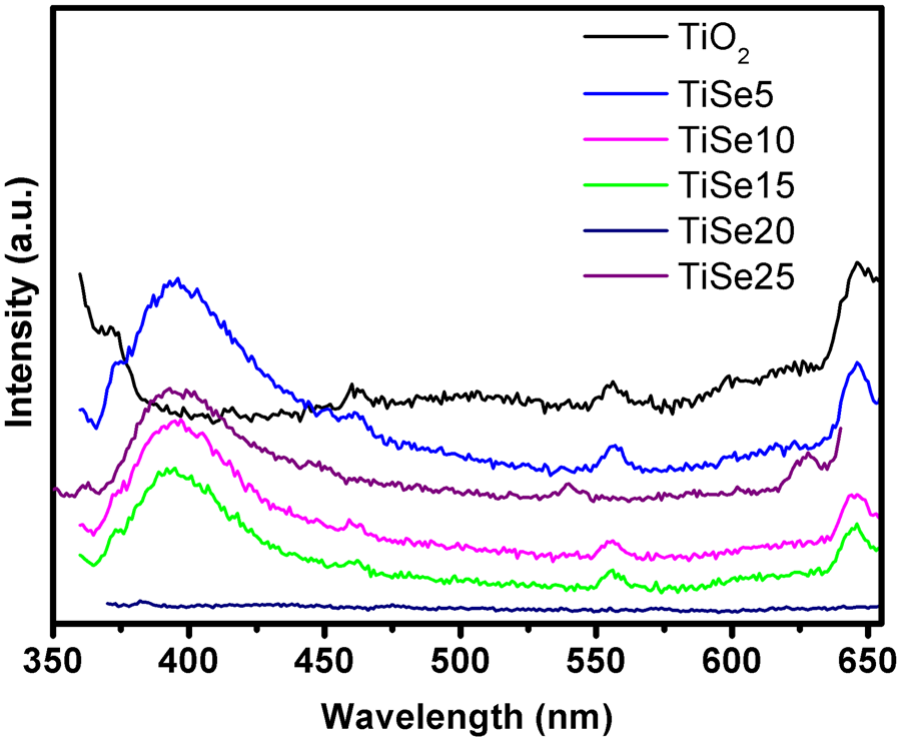
PL spectra of Se-doped TiO_2_.

PL spectra have been widely used to investigate the change of surface states of TiO_2_, the efficiency of charge carrier trapping, immigration and transfer to understand the fate of electron-hole pairs in semiconductor particles^39^. Figure 5 shows the PL spectra of Se-doped TiO_2_. A strong peak at 394 nm can be found for Se-doped TiO_2_, but lacking in TiO_2_, indicating the significantly suppressed electron-hole recombination in TiO_2_, which might be due to the high defects concentration^40^. Slight Se doping improves the crystalline structure, which may suppress the defects concentration and increase the electron-hole recombination rate. Clear PL peaks can be observed in Se-doped TiO_2_. However, with increasing Se doping concentration, more defects are formed, which provides more electron trapping centers. Interestingly, no PL peaks can be observed for TSe20, suggesting the strongly suppressed electron-hole recombination rate^40^. Further increasing Se doping concentration to TSe25, strong PL peak can be observed again. This indicates that there must be an optimum level up to which the addition of doping ions can help to lower electron-hole recombination rate. Otherwise, the excess doping ions act as recombination centers, which can dramatically decrease the photocatalytic activity of the doped catalyst^27^.

**Figure 6:**
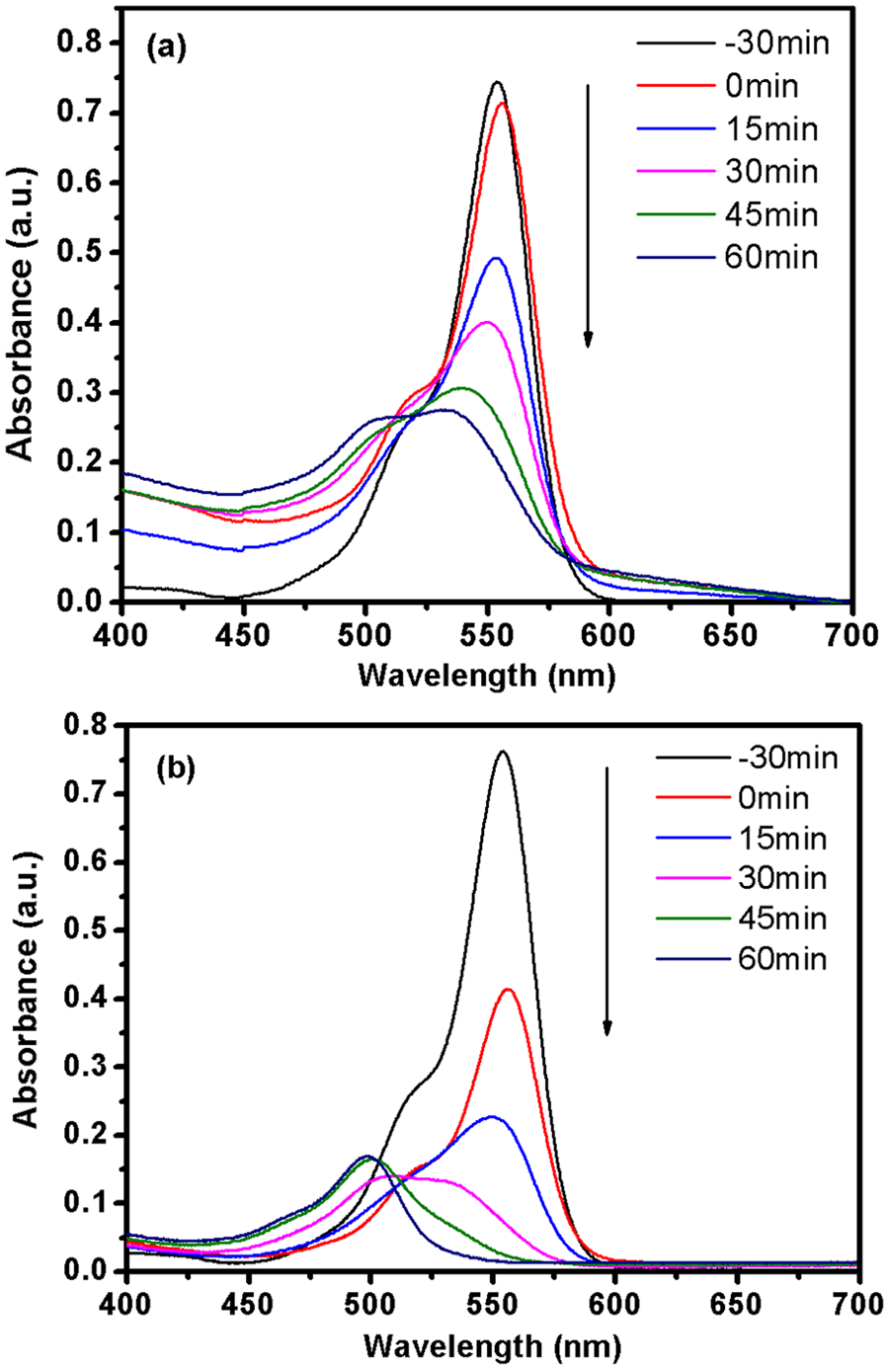
UV-Vis absorption spectra changes of RhB solutions by (**a**) TiO_2_ and (**b**) TSe20 at various times.

The typical time-dependent UV-Vis spectra of RhB solution by TiO_2_ and TSe20 in photochemical reaction are shown in Fig. 6. The intensity of the characteristic absorption peak of RhB solution decreases with time. It can be seen that TiO_2_ can effectively decompose RhB under the visible light irradiation. TSe20 shows much faster decomposition of RhB under visible irradiation, confirming the promotion of Sedoping on the photocatalytic activity of TiO_2_. The absorption peak position shifts to shorter wavelength, revealing that RhB is de-ethylated in a stepwise manner (i.e., ethyl groups are removed one by one as confirmed by the gradual peak wavelength shifts toward the blue region)^41^. However, after 45 minute, for RhB under irradiation in the presence of TSe20, the shift of wavelength reaches maximum. This indicates the decomposition mechanism of RhB changes to destroy its conjugated structure^42^.

**Figure 7:**
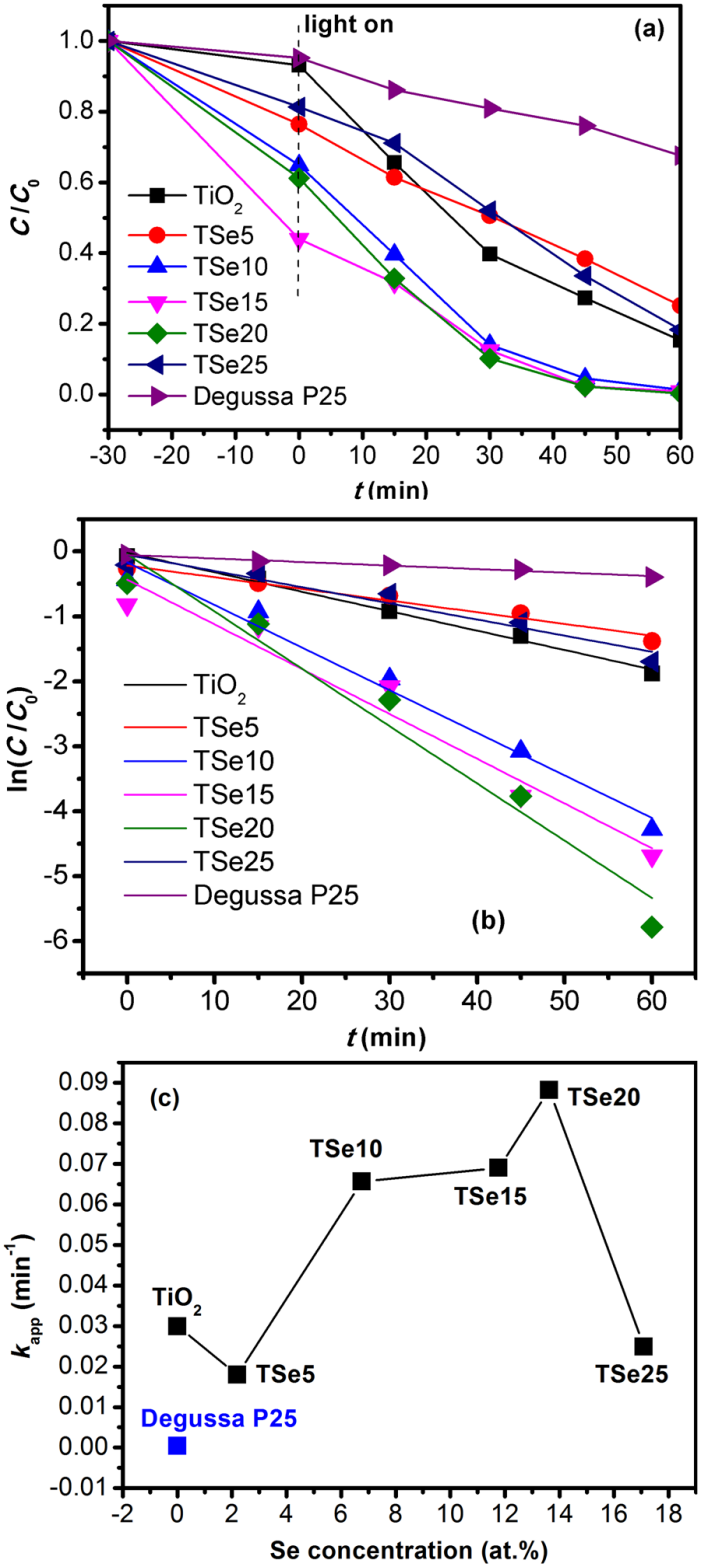
(**a**) Photo-decolonization ratios of RhB under visible light irradiation, (**b**) first order kinetics fitting using ln(*C*_0_/*C*)-*t*, (**c**) the catalytic reaction rate of each sample.

Photocatalytic reactions for the degradation of RhB aqueous solutions using Se-doped TiO_2_ are shown in Fig. 7. For comparison, the photocatalytic activity of Degussa P25 was also measured. For the first 30 min in dark, it can be seen that Se-doped TiO_2_ shows higher adsorption ability than TiO_2_. Since the crystallite size of all the samples is comparable, the surface area should not be the main reason. It is supposed to be due to more active sites on the surface of Se-doped TiO_2_^43^. TiO_2_ shows superior photocatalytic performance than Degussa P25. With Se doping, the photocatalytic capability is significantly improved. After irradiation for 30 min, nearly 91.3% of RhB was degraded by the sample TSe20, while only 60.3% Rhb was degraded by TiO_2_. Experimental dependencies of the molar concentration of RhB in the presence of undoped/doped TiO_2_ and Degussa P25 powders in visible irradiation exhibit pseudo first order kinetics, as shown in Fig. 7(b) by plotting ln(*C*/*C*o) versus irradiation time, *t*. The apparent reaction rate constant (*k*_app_) is calculated from the slope of the curve, as illustrated in Fig. 7(c). With small concentration of Se doping, TSe5 shows much smaller *k*_app_ value. With further increasing Se doping concentration, *k*_app_ continuously increases. TSe20 exhibits the highest *k*_app_. With further increasing Se doping concentration, *k*_app_ of TSe25 drastically decreases. Comparing the rate constant values under the visible light irradiations, the powder TSe20 attains the highest *k*_app_ value of 0.088 min^−1^, which is much higher than that of undoped TiO_2_ (0.030 min^−1^) and Degussa P25 (0.0004 min^−1^). TiO_2_ exhibits better photocatalytic activity than Degussa P25, mainly because of the synergetic effect of narrow band gap in the visible light range and mixed phases (orthorhombic and anatase) which hinder the recombination of generated electron-hole pairs^44^.

**Figure 8:**
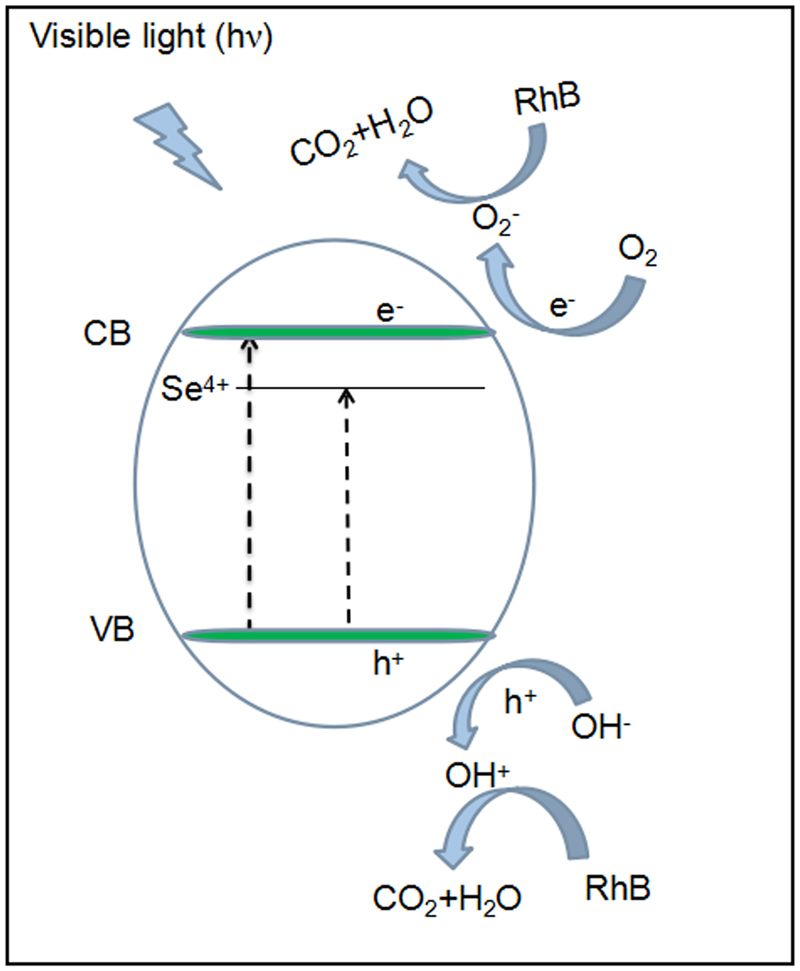
Schematic diagram of mechanism involved in the photocatalytic degradation of RhB by Se-doped TiO_2_.

As shown in Fig. 8, the photoinduced electrons and holes play important role in the photocatalytic reaction. The electrons are adsorbed by O_2_ to produce superoxide radical anions of ·O_2_^−^, while holes can oxidize OH^−^ to produce ·OH. Both can further react with RhB which is decomposed to CO_2_ and H_2_O. The improved photocatalytic activity of Se-doped TiO_2_ under visible light can be first understood by the decreasing band gap into the visible range with increasing Se doping concentration, which might increase the absorption efficiency of the visible light, as shown in Fig. 8. It has been calculated that Se^4+^ doping into TiO_2_ may induce additional mid-gap electronic levels close to the conduction band edge^24^. These intermediate electronic states are determined to be mainly originated from the Se 3p states and not populated by electrons. They are not donor states but allowed energy states hybridized with the Ti 3d states in the conduction band. The increase in the concentration of dopant Se^4+^ introduces more electronic states into the band-gap, enhancing the density of electronic states in the gap. These intermediate energy levels offer additional steps for the absorption of low energy photons through the excitation of valence band electrons to these intermediate energy levels, from where they can be excited again to the conduction band. Furthermore, the photocatalytic capability also depends on the separation efficiency of generated electron-hole pairs. To improve the photocatalytic activity, the photoinduced electrons and holes inside the particles should survive during the transportation to the surface of particles, and the recombination should be avoided. As can be seen, no obvious peaks can be observed in the PL spectrum of TSe20, indicating the strongly suppressed recombination rate of electron-hole pairs. The defects, such as O vacancies, Ti^3+^, etc. may play significant role, since they can trap the electrons and suppress the recombination of electron-hole pairs^27^. With slight concentration of Se doping, the crystal structure is improved, thus defects concentration is decreased, leading to the worse photocatalytic capability for TSe5. With further increasing Se doping concentration, more defects will be introduced in Se-doped TiO_2_, the photo-generated electrons can be effectively trapped, and the recombination of electron-hole pairs is effectively suppressed, leading to the improved photocatalytic performance. As can be seen from PL measurements, TSe20 exhibits the negligible PL intensity, indicating the lowest recombination rate of electron-hole pairs. Together with the band gap in visible range, TSe20 exhibits the best photocatalytic performance. With further increasing the Se doping concentration beyond this optimum value, the average distance between the trapping sites is so small that large amount of them will be confined within the crystal lattice, increasing the recombination of electron-hole pairs, as can be seen the significant PL intensity for TSe25^27^. The photocatalytic performance of TSe25 decreases drastically. Thus, it can be concluded that the improved photocatalytic activity in Se-doped TiO_2_ with optimum concentration (TSe20) is a synergetic contribution from the narrow band gap by Se^4+^ doping and suppressed electron-hole pairs recombination due to the optimum defects concentration.

## Conclusions

A series of Se-doped TiO_2_ nanoparticles of anatase structure with various Se concentrations up to 17.1 at.% were prepared using sol-gel method. Slight concentration of Se doping has been confirmed to improve the crystalline structure of TiO_2_, while higher concentration of Se doping deteriorates the crystalline structure. The doped Se ions are confirmed to be mainly in the valence state of + 4, which provides extra electronic states in the band gap of TiO_2_. The band gap is effectively narrowed with the smallest gap energy of 2.17 eV by Se doping of concentration of 6.76 at.%. With further increasing Se doping concentration, Se doping significantly improves the photocatalytic activity of TiO_2_ and 13.63 at.% Se-doped TiO_2_ has the highest photocatalytic activity from the photo degradation of RhB in aqueous solution under visible light irradiation, which is attributed to the synergetic contribution of narrowed band gap by Se doping and suppressed electron-hole recombination due to the optimum defect density.

## Electronic supplementary material

Supplementary materials
