# Supplementary material for: Enhanced photocatalytic activity of Se-doped TiO2 under visible light irradiation
Source: Sci Rep. 2018 Jun 8;8:8752. doi: 10.1038/s41598-018-27135-4 (PMC5993730; doi:10.1038/s41598-018-27135-4)
Supplement: Supplementary file 1 — Supplementary materials [file 41598_2018_27135_MOESM1_ESM.docx]

**Supplementary materials:**

**Enhanced** **photocatalytic activity of Se-doped TiO_2_ under** **visible light irradiation**

Wei Xie, Rui Li, Qingyu Xu^*^

Yangzhou Polytechnic Institute, Yangzhou, Jiangsu, China

Corresponding author: xuqingyu@seu.edu.cn


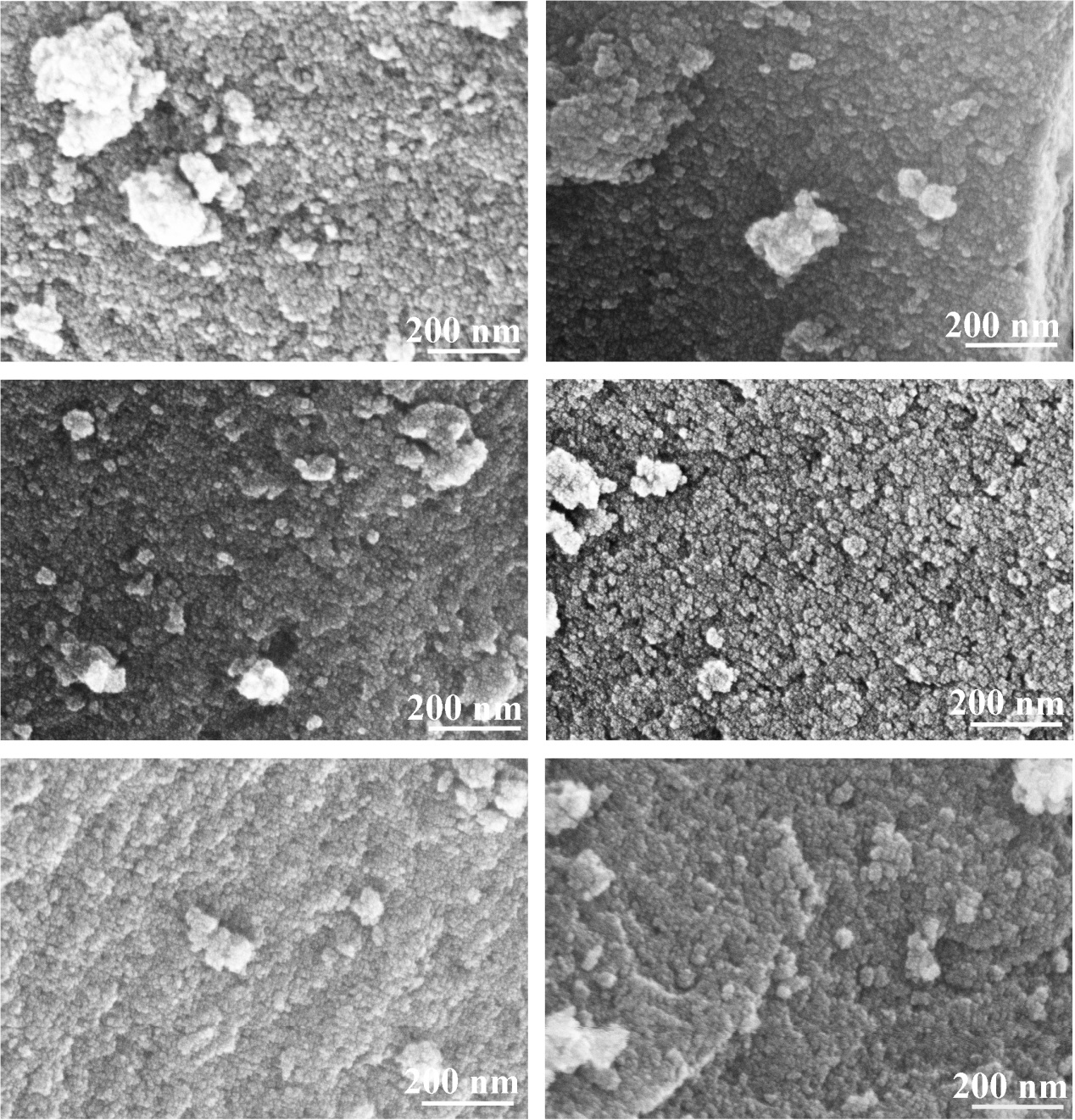
Fig. S1 SEM image of (a) TiO_2_, (b) TSe5, (c) TSe10, (d) TSe15, (e) TSe20, (f) TSe25.

Fig. S2 Photoluminescence excitation (PLE) spectra of TiO_2_, TSe5, TSe10, TSe15, TSe20, TSe25 for the PL emission at 430 nm.


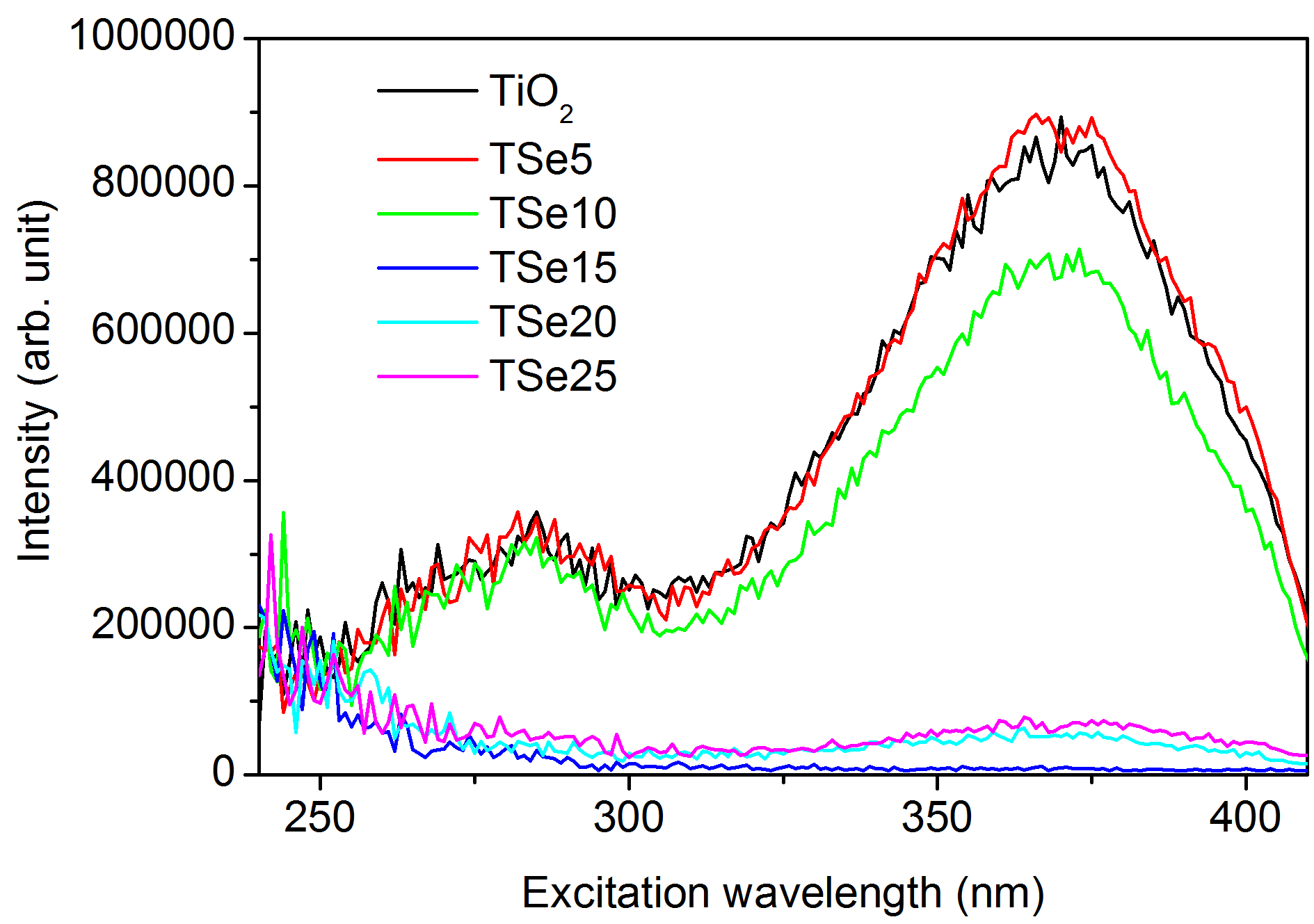


PLE might be applied to determine the band gap [1]. The excitation peaks not only come from the energy levels of host lattice, but also the energy levels of doped ions [2]. Since we don’t observe PL peaks related to the energy levels of doped Se ions, we cannot do the PLE measurement from the corresponding PL emission. Compared with the PLE spectrum of TiO_2_, the excitation peaks from the host lattice of TiO_2_ continuously decrease with increasing Se doping concentration. This gives an indirect evidence of the replacement of energy levels from doped Se ions.

References:

[1] H. Nakajima, T. Mori, Q. Shen, T. Toyoda, Chem. Phys. Lett. 409, 81 (2005).

[2] Y. Shimizu, K. Ueda, Y. Inaguma, Opt. Mater. 66, 327 (2017).
